# Robust performances of a nocturnal long-term ECG algorithm for the evaluation of sleep apnea syndrome: A pilot study

**DOI:** 10.1371/journal.pone.0318622

**Published:** 2025-05-16

**Authors:** Pauline Guyot, Morgane Eveilleau, Thierry Bastogne, Carole Ayav, Nicolas Carpentier, Bruno Chenuel

**Affiliations:** 1 NOVIGA, Nancy, France; 2 CRAN UMR 7039, Université de Lorraine, CNRS, Vandœuvre-lès-Nancy, France; 3 Clinical Epidemiology Centre CIC-1433, CHRU Nancy, Inserm, Vandoeuvre-lès-Nancy, France; 4 Centre de Médecine et de Recherche sur le Sommeil, Service de Neurologie, CHRU Nancy, Nancy, France; 5 CHRU Nancy, Hôpitaux de Brabois, Nancy, France; 6 EA 3450 DevAH, Université de Lorraine, Vandœuvre-lès-Nancy, France; Baruch Padeh Medical Center Poriya, ISRAEL

## Abstract

Obstructive sleep apnea-hypopnea syndrome (OSAHS) is one of the most common sleep disorders affecting nearly one billion of the global adult population, making it a major public health issue. Even if in-lab polysomnography (PSG) remains the gold standard to diagnose OSAHS, there is a growing interest to develop new solutions with more convenient at home devices enhanced with AI-based algorithms for the detection of sleep apnea. This retrospective study aimed to assess the performances of a new method based on nocturnal long-term electrocardiogram signal to detect apneas and hypopneas, in patients who performed attended in-lab PSG. After assessing the quality of the ECG signal, the new method automatically detected apneas and hypopneas using dedicated machine learning algorithm. The agreement between the new ECG-based detection method and the standard interpretation of PSG by a sleep clinician was determined in a blind manner. Eighty-five exams were included into the study with a mean bias between the proposed method and the scorer of 3.5 apneas-hypopneas/hour (/h) (95% CI -48.1 to 55.1). At a threshold of 15/h, sensibility and specificity were 93.3% and 66.7% respectively, and positive and negative predictive values were 87.5% and 80%, respectively. The proposed method using nocturnal long-term electrocardiogram signals showed very high performances to detect apneas and hypopneas. Its implementation in a simple ECG-based device would offer a promising opportunity for preliminary evaluation of patients suspected or at-risk of OSAHS.

## Introduction

Obstructive Sleep Apnea-Hypopnea Syndrome (OSAHS) is one of the most common sleep-related breathing disorder with a prevalence ranging from 2% to 14% in the adult general population [1]. Sleep-related obstructive breathing events consist of recurrent and transient breathing arrests (apneas) or decreased respiratory flows (hypopneas), caused by episodes of complete or partial closure of the upper airways during sleep, associated with reductions of blood oxygen contents and/or micro-arousals [2]. The average number of apneas and hypopneas per hour of sleep (apnea-hypopnea index, AHI) is the most used indicator of the OSAHS severity and major studies in the field reported a dose-effect relationship between AHI and cardiovascular outcomes, especially incidental myocardial infarction and stroke [3]. OSAHS may also entail lifelong medical conditions as various as renal, chronic arterial hypertension, irregular heart rhythms, hepatic complications and also excessive daytime and impaired quality of life [4]. All in it, the burden of OSAHS in the United States of America may reach the value of 130 billion dollars a year [5]. While the prevalence of sleep apnea in the population consulting a cardiologist is high (estimated at 40–50% of patients with hypertension and at 30% of patients with coronary artery disease), there is no systematic screening tool for this medical specialty [6]. As the continuous positive airway pressure (CPAP) allows reversing the cardiovascular risk related to OSAHS [7], it is crucial to improve the capacities for diagnosis.

Polysomnography (PSG) is internationally recognized as the gold standard exam for diagnosing sleep disorders including OSAHS since it remains the unique tool to estimate AHI with precision. PSG includes the measurement of numerous physiological variables such as electroencephalogram (EEG), electrooculogram (EOG), chin and legs electromyograms (EMG), transcutaneous oxygen saturation, oronasal airflow and the concomitant respiratory work through thoraco-abdominal enlargement, electrocardiogram (ECG) and body position [8]. Oxygen saturation, respiratory flow and effort are necessary for counting sleep-related events (numerator of AHI), whereas EEG (together with EOG and chin EMG) is essential to identify both micro-arousals for counting additional hypopneas based on composite criteria, and above all the total sleep time, giving a true value of the AHI denominator. However, PSG is a time-consuming and expensive exam with a lengthy waiting time that limits its accessibility to the entire population at risk [9].

Several alternative solutions have been developing to search for sleep apneas both in-lab or in patients’ homes. They embed both fewer sensors and analysis algorithms dedicated to the AHI estimation. The American Academy of Sleep Medicine (AASM) categorized four levels of sleep study devices according to the exhaustivity of data recording [9]. In this classification, the type I and type II categories correspond to a full-channel PSG in a sleep laboratory with or without a sleep technician in attendance respectively. The type III referred to simplified home devices based on at least four types of measurement, two of which relate to the oro-nasal airflow coupled with oximetry and ECG/or heart rate measurement. The type IV of this classification referred to home devices recording one or two respiratory channels. Giving the size of the population at risk for OSAHS, there is a growing need for developing ever better tools for diagnosis. The ideal solution shall gather key features from type III-IV devices like accessibility, user-friendliness for both patients and clinicians, and minimal costs, together with diagnosis precision and reliability related to type I-II categories.

One of the promising approaches to identify OSAHS is based on ECG analysis. Sleep-disordered breathing induces dramatic changes in heart rate (HR) and in hemodynamics [10,11]. More particularly, episodes of obstructive sleep apneas produce repetitive inspiratory efforts leading to highly negative intrathoracic swings and transient decreases in left ventricular stroke volume [12]. Then, cardiac output falls during obstructive apnea, secondary to decreased stroke volume and also to reductions in heart rate [13]. The consequent heart rate pattern known as Cyclic Variation of Heart Rate (CVHR) is marked by intermittent fluctuations in HR: a progressive bradycardia is observed at the onset of apnea, followed by a sudden tachycardia during the arousal phase [14]. In addition to these periodic changes, OSAHS is linked to electrical myocardial remodeling, including a reduction in QRS voltage and an increased prevalence of QRS fragmentation [15]. Also, a leftward shift of the electrical axis is frequently observed and is significantly associated with the severity of sleep apnea syndrome [15].

In this retrospective clinical study, we tested a new ECG lead-based method of apnea-hypopnea detection among a sample of in-lab patients suspected of sleep apnea syndrome (SAS). The main objective was to assess the concordance between a new ECG-based detection algorithm and the standard interpretation of PSG by a sleep clinician for the evaluation of sleep apnea syndrome.

## Methods

### Subjects

We carried out a monocentric retrospective clinical study. All patients screened were admitted to the Center for Medicine and Research in Sleep in Nancy University Hospital for the diagnosis of sleep apnea. The selection criteria were based on the value of apnea-hypopnea index (AHI) in order to form a harmonious distribution of the number of subjects in each class of interest. We set three classes of interest: mild AHI (5/h ≤ AHI < 15/h), moderate AHI (15/h ≤ AHI < 30/h) and severe AHI (AHI ≥ 30/h). We estimated *a priori* a total number of 150 subjects to analyze, with 50 patients per class of interest and at best, equally involving obstructive and central apneas. Patients were included consecutively from January 1^st^ 2019 to July 1^st^ 2020. The local ethical committee approved the study protocol (“saisine n°280 du Comité d’Éthique du CHRU de Nancy”) and all patients had provided their written consent to participate before inclusion.

### Sleep data collection

Polysomnography (PSG) included seven electroencephalogram (EEG) leads C3, C4, O1, O2, T3, T4, and Fz as the reference according to the 10/20 system (impedance < 5kΩ). Two bipolar electrodes respectively placed 1 cm below the left outer canthus (EOG 1) and 1 cm above the right outer canthus (EOG 2) recorded the electrooculogram (EOG). Two chin bipolar electrodes recorded the submental electromyogram (EMG). Myographic recordings also included two bipolar left and right anterior tibialis EMG. Cardiorespiratory parameters included a bipolar ECG (DII), one nasal pressure sensor, one oral thermistor, one transcutaneous oximeter, thoracic and abdominal strain gauges. Recordings were acquired using an in-lab multichannel device at a 400 Hz sampling rate (B3IP©, Medatec©, Belgium).

### PSG-based sleep scoring

Sleep was scored according to the AASM international standard rules, revised in 2017 [16]. All recordings were visually scored by a trained sleep clinician (NC). Hypopnea was defined as a ≥  30% decrease of nasal and/or buccal airflow during ≥  10s associated with a ≥  3% decrease of transcutaneous hemoglobin saturation (desaturation) and/or a micro-arousal. Apnea was defined as a ≥  90% decrease of nasal and/or buccal airflow during ≥  10s. Apnea was classified as obstructive apnea if the event was associated with an increase of inspiratory work based on abdomino-thoracic curves, whereas apnea was defined as central, if the event was associated with a lack of inspiratory work. AHI corresponds to the ratio of the number of apneas and hypopneas to the total sleep time (TST) per hours. In the cases of AHI ≥ 15/h, if the ratio of central events [number of central apneas/(number of apneas +  hypopneas)] was >  0.5, sleep apnea syndrome was qualified as central. In other cases, sleep apnea syndrome was defined as obstructive. To limit misclassifications of sleep apnea syndrome, hypopneas were not qualified as obstructive nor central. Patients were qualified as apnea-predominant when AI ≥  HI or hypopnea-predominant when HI>  AI, both with an AHI superior or equal to 15/h (AI: Apnea Index, HI: Hypopnea Index). For patients with multiple nights, only the last one was selected to limit the first night effect.

### ECG processing

**Fig 1:**
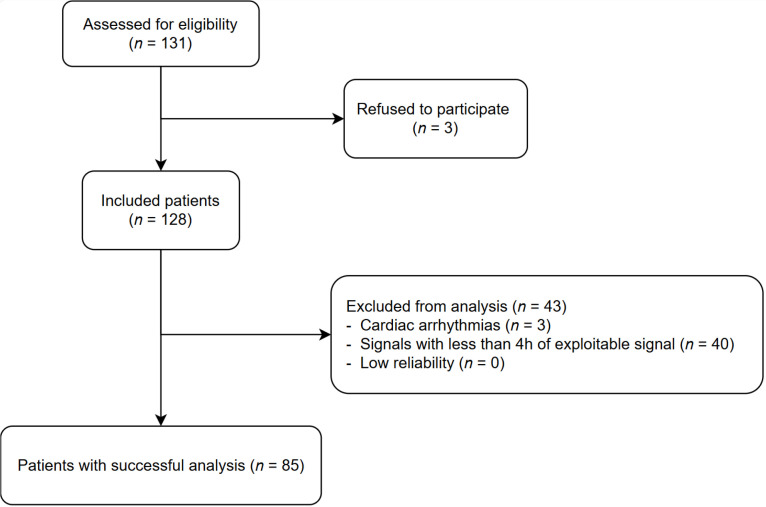
ECG lead-based algorithm diagram. sECG: raw ECG signal, s^d^ECG: denoised ECG signal, (z_ts_, z_te_): start and end of good quality signal zones, (z^n^_ts_, z^n^_te_): start and end of good quality signal combined with normal cardiac rhythm zones, ^s^EDR: EDR series, (e_ts_, e_te_, c): start, end and class of abnormal events (apnea or hypopnea), TST: total sleep time, AHI: apnea-hypopnea index.

**Fig 2:**
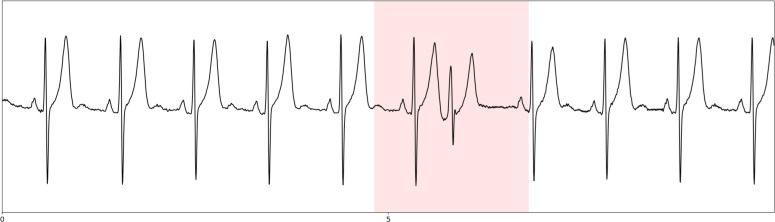
Features of interest detected or computed on an ECG signal. R peak, QRS_onset_ and QRS_offset_ are key points in an ECG beat, whereas QRS height, QRS width and RR interval are computed using the previous key points.

The ECG signal analysis was divided into seven successive steps, as illustrated in Fig 1. In Fig 2, the different features of interest used in the ECG signal are described for better comprehension.

#### Signal denoising.

**Fig 3:**
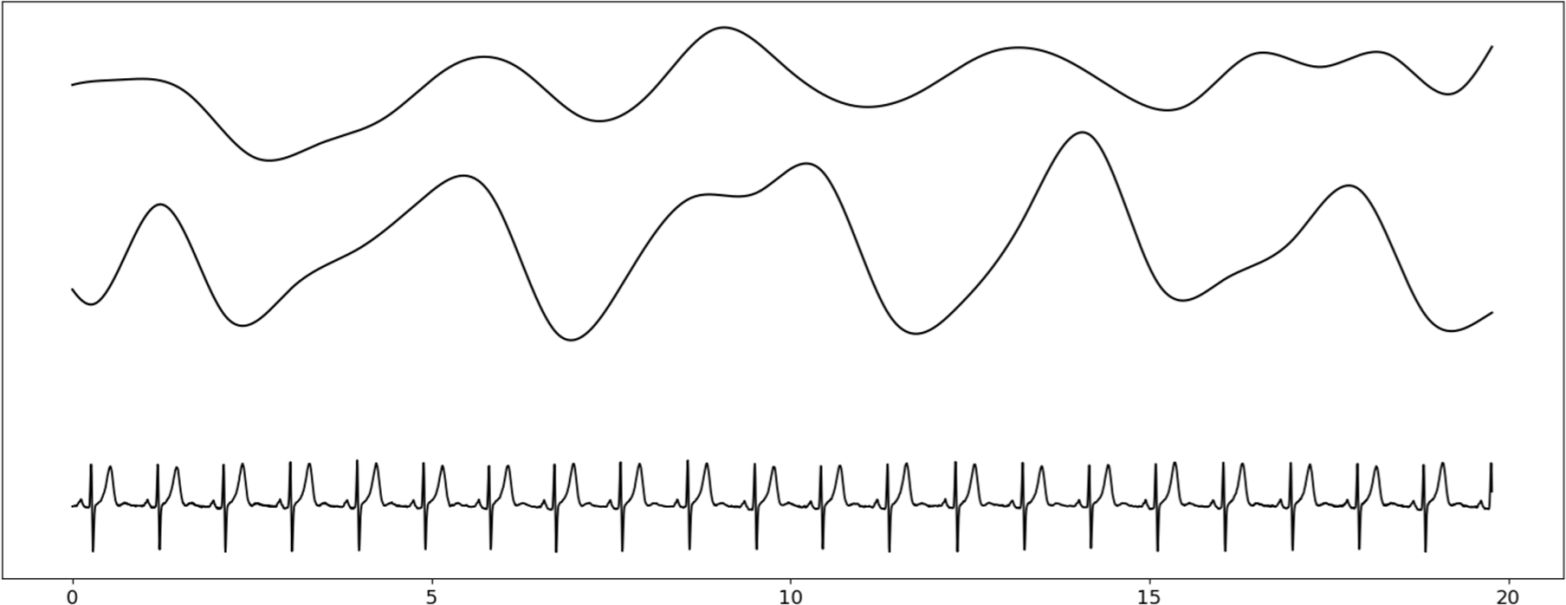
Signal denoising (baseline correction and powerline noise removal) example. Top: raw ECG signal in black with baseline wander in red; down: denoised ECG signal. X-axis is the time scale in seconds.

Signal was resampled at 250Hz, a median filtering of 0.2s for baseline correction [17] and a band-stop filtering with a cut-off frequency of 50Hz for powerline noise removal were applied (an example of signal denoising is presented in Fig 3).

#### Quality assessment.

**Fig 4:**
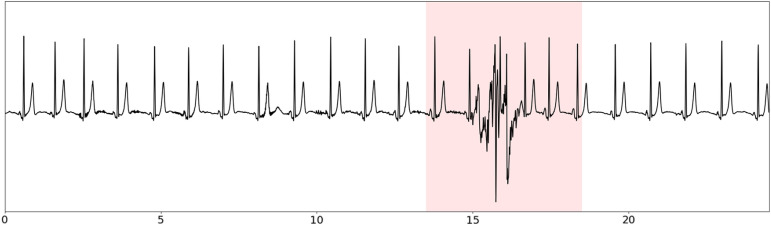
Suppression of low-quality zone example. In red, the detected noisy zone to be suppressed for the rest of the analysis. X-axis is the time scale in seconds.

This step consisted of detecting unhooked or saturated electrodes but also parts of the signals that may be too noisy to be processed. Kurtosis-based method using sliding windows of 5 seconds was used [18]. A threshold was determined above which the signal was considered low-quality, meaning QRS complexes were hardly detected. Only good quality portions were kept. An example of low-quality zone detection is presented in Fig 4.

#### Selection of epochs of regular cardiac rhythm.

**Fig 5:**
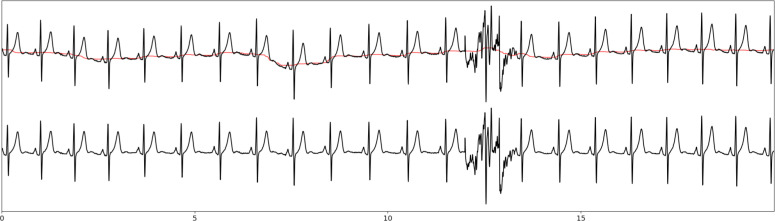
Detection of an abnormal beat example. In red, an abnormal beat zone to be suppressed for the rest of the analysis. X-axis is the time scale in seconds.

This step aimed to detect and exclude epochs containing cardiac arrhythmias from analysis. First of all, R-peaks, QRS onsets and offsets were detected using *Orthogonal Matching Pursuit* (OMP) algorithm with a dedicated dictionary [19]. We considered four ECG features to detect the presence of cardiac arrhythmias: QRS width, height and form, and heart rate. For example, a simplified detection of ectopic beat was realized using the following criteria: (a) QRS width longer than 200ms, (b) RR interval longer than 1.2s or shorter than 0.6s, (c) QRS height higher than mean of 10 previous QRS complexes. Portions of signals with no arrhythmia were kept. An example of abnormal beat detection is presented in Fig 5.

As the AASM recommends a minimum of four hours of good quality sleep data when a home sleep apnea testing is performed [20], we only kept for analysis exams cumulating four hours of signal both with good quality and without cardiac arrhythmia.

#### EDR series extraction.

**Fig 6:**
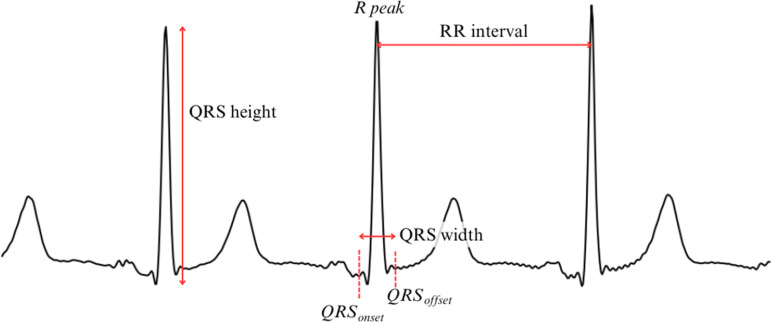
Example of EDR series extracted from ECG signal. From top to bottom: HRV series, RWA series and denoised ECG signal. X-axis is the time scale in seconds.

The goal of this step is to obtain a reconstructed respiration signal from ECG. ECG Derived Respiration (EDR) series were extracted from the ECG with regular cardiac rhythm. Several EDR series were calculated, among them Heart-Rate Variability (HRV), R-Wave Amplitude (RWA), and other beat-related morphology. An example of EDR series is displayed in Fig 6.

#### Classification normal/ abnormal breathing.

The goal of this step is to distinguish between normal breathing and pathological events, and to characterize those events between apnea and hypopnea. Using those EDR series, statistical and morphological parameters were computed to assess respiratory effort. Those parameters were used as inputs in an agglomerative hierarchical clustering to detect normal and abnormal breathing events.

Then, analysis of EDR series before and after each suspected event allowed to confirm the presence of a pathological event and its type (apnea or hypopnea).

#### Classification wake/ sleep.

In parallel to the detection of normal and abnormal breathing, the TST was calculated. Sleep/wake classification was performed using a convolutional neural network using 30-seconds epoch of EDR series as inputs. Training was realized using public databases such as St. Vincent’s University Hospital/ University College Dublin Sleep Apnea and MIT-BIH Polysomnographic [21,22].

#### AHI and reliability index estimation.

This final step aims at computing AHI and the reliability index. Using the outputs of the previous classification steps, AHI is calculated from the number of detected abnormal respiratory events and the TST. As the proposed method is fully automated, a reliability index was proposed. The latter took two parameters into account:

(i) the percentage of sleep time analyzed by the algorithm compared to total sleep time;
(ii) the relevance of the results provided by the algorithm by examining the resemblance of the case examined with those used during the learning phase.

The closer the relevance is to 100, the more the detected events resemble to what the algorithm has learned and the more the percentage of analyzed sleep time is high. The closer the relevance is to 100, the more reliable the results are. This index can take three colors: grey for results not provided, orange for results with moderate uncertainty and green for greater confidence in the results (Table 1).

**Table 1:** Determination of reliability index.

		Relevance of results		
		0-50%	50-70%	70-100%
% TST	0-50%			
	50-100%			

TST: Total Sleep Time.

As retrospective data were used, sensors placement was not checked before signal acquisition. The morphology of QRS complex (monophasic or biphasic) was computed to evaluate its impact on apnea detection. Monophasic QRS complex, i.e., the amplitude of the largest positive or the deepest negative wave is bigger than the other one, is the most common morphology regarding DII lead. Biphasic QRS complex, i.e., the amplitudes of the largest positive and deepest negative waves are similar, may be linked to patient-related cardiac electrical axis (right axis, DII), but also to electrodes misplacement (orthogonal placement of DII from cardiac axis) or even electrodes inversion (inverted DII polarity).

### Statistical analysis

Statistical tests were 2-sided with a statistical significance at.05. Firstly, Shapiro-Wilk test was used to test for normality for each parameter and comparison between population were completed using t-test for sample means in the case of normal distribution, and using Wilcoxon rank sum test for sample medians in the case non-normal distribution.

Average bias and its 95% limits of agreement are also calculated as follows:


$$b=\frac{1}{N}\overset{N}{\underset{i=1}{\sum }}(AHI_{PSG}^{i}-AHI_{ECG}^{i})$$ (1)

$$\{\begin{array}{c} l_{h}=b+1.96\sigma \\ l_{b}=b-1.96\sigma \end{array}$$ (2)

with $AHI_{PSG}\in R^{N}$ the AHI estimated by polysomnography, $AHI_{ECG}\in R^{N}$ the AHI estimated by the ECG-based detection algorithm, *N* the number of analyzed exams, *σ* the standard deviation of $AHI_{PSG}-AHI_{ECG}$, *b* the bias and $\left(l_{h},l_{l}\right)$ limits of agreements respectively high and low. Bland-Altman analysis serves to describe agreement between two quantitative measurements using mean and difference of both populations.

Classic performance classification metrics were used using specificity (Sp), sensitivity (Se), positive and negative predictive values (PPV and NPV), accuracy (Acc) and F1-score (F1) at a fixed threshold. All these parameters are computed as follows:


$$Se=\frac{TP}{TP+FN}$$ (3)

$$PPV=\frac{TP}{TP+FP}$$ (4)

$$Acc=\frac{TP+TN}{TP+FP+TN+FN}$$ (5)

$$Sp=\frac{TN}{TN+FP}$$ (6)

$$NPV=\frac{TN}{TN+FN}$$ (7)

$$F1-score=2*\frac{PPV*Se}{PPV+Se}$$ (8)

with TP, FP, TN, FN are respectively the number of true positives, false positives, true negatives, and false negatives.

All statistical analysis was performed using Python (version 3.9) in Spyder software (version 5.1.1, Scipy library).

## Results

### Patients and PSG results

**Fig 7:**
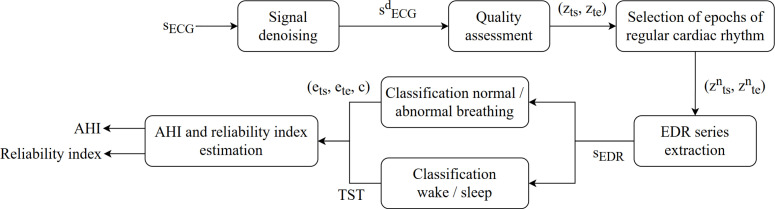
Flow chart of study. n: number of patients.

As detailed in Fig 7, 131 patients were eligible for the trial among them 3 refused to participate, giving 128 patients assessed for analysis. Among those patients, 85 had a complete successful analysis. Demographic characteristics of the 128 included patients and the 85 patients with successful analysis are summarized in Table 2.

**Table 2:** Demographic characteristics of the patients assessed for analysis (n = 128) and the patients with successful analysis (n = 85).

Characteristics	Included patients		Patients with successful analysis		Test p-value
Sex (female)	52	40,63%	36	42,35%	p = 0.71
Age (y)	53,58	19,05	57,62	16,56	p = 0.0025
BMI (kg/m^2^)	26,56	4,89	27,08	4,99	p = 0.08
AHI by PSG (evts/h)	22,06	19,91	25,88	21,42	p = 0.14
Apnea predominance	21	16,40%	15	17,64%	p = 0.76
Total sleep time (h)	7,12	2,20	6,92	2,03	p = 0.18

Data presented as mean (SD) or n (%). BMI: Body Mass Index, AHI: Apnea-Hypopnea Index.

The main source of exclusion was insufficient length of exploitable signal ( < 4 hours), which can be explained by too much noise in the electrocardiogram signal. Except for the age, there was no significant difference in the repartition of patients in terms of demographic characteristics, severity classes and mechanism of apnea syndrome between the patients assessed for analysis and the patients with successful analysis, as showed in Tables 2 and 3.

**Table 3:** Repartition of included patients (n = 128) and patients with successful analysis (n = 85) in severity classes and mechanism of the syndrome.

	Included patients (n = 128)				Patients with successful analysis (n = 85)			
	<15/h	15-30/h	≥30/h	Total	<15/h	15-30/h	≥30/h	Total
Mild or no SAS	54	0	0	54	32	0	0	32
Obstructive sleep apnea	0	31	35	66	0	22	25	47
Central sleep apnea	0	4	4	8	0	4	2	6
Total	54	35	39	**128**	**32**	**26**	**27**	**85**

Chi-2 test p-value: p =  0.59, no significant difference between the two populations*.*

### ECG-based detection algorithm vs PSG: respiratory events (AHI)

A Wilcoxon rank sum test for the difference between median AHI of the PSG and of the ECG-based detection algorithm was performed as the AHI was not distributed normally and gave a non-statistically significant result with a p-value of 0.17. The bias between AHI of the PSG and of the ECG-based detection algorithm was of 3.5/h with limits of agreement from -48.1 to 55.1/h. Results are presented in Table 4.

**Table 4:** Bias value of AHI between polysomnography and the ECG-based detection algorithm for different classes of: (i) severity of AHI, (ii) reliability index.

	Total dataset	Total dataset			Good reliability (green)			Moderate reliability (orange)		
		0-15/h	15-30/h	≥30/h	0-15/h	15-30/h	≥30/h	0-15/h	15-30/h	≥30/h
n	85	32	26	27	21	15	18	11	11	9
Bias (/h)	3.5	-10.7	-5.0	28.5	-2.0	-0.3	34.5	-27.2	-11.4	16.6
Limits of agreement (/h)	[-48.1; + 55.1]	[-50.1; + 28.7]	[-39.2; + 29.2]	[-13.9; + 71.1]	[-35.43; + 31.34]	[-41.25; + 40.72]	[-8.86; + 77.96]	[-55.2; + 0.7]	[-25.6; + 2.7]	[-12.3; + 45.5]
Standard deviation	26.3	20.1	17.4	21.7	17.0	20.9	22.1	14.3	7.1	14.7

n: number of exams in the class.

For exams with good reliability index, bias is of -2.0/h for no or mild SAS, -0.3/h for moderate SAS and 34.5/h for severe SAS. For exams with moderate reliability index, bias is of -27.2/h for no or mild SAS, -11.4/h for moderate SAS and of 16.6/h for severe SAS. Bias is also computed respectively according to the mechanism of the respiratory events (obstructive or central) and predominance of the type of events (hypopneas if hypopneas>apneas, else apneas). Results are presented in Table 5.

**Table 5:** Bias value of AHI between polysomnography and the ECG-based detection algorithm for different classes of: (i) predominance of the type of events (hypopneas or apneas), (ii) main mechanism involved in the majority of apneas genesis (obstructive or central).

	Predominance		Mechanism	
	Hypopnea (HI>AI)	Apnea (AI≥HI)	Obstructive	Central
n	38	15	47	6
Bias (/h)	14.9	10.9	11.3	12.2
Limits of agreement (/h)	[-37; + 66.8]	[-39.1; + 61.1]	[-30.8; 53.4]	[-39.6; 63.9]
Standard deviation	26.4	25.5	21.5	26.4

n: number of exams in the class.

Detection performances are presented in Table 6 for total dataset, exams with good reliability and different QRS morphology classes.

**Table 6:** Performance values of ECG-based detection algorithm at an AHI threshold of 15/h compared to gold standard polysomnography.

	Total dataset	Good reliability		
		All morphologies	Biphasic QRS	Monophasic QRS
n	85	54	25	29
Se	69,8%	78,5%	63,6%	93,3%
Sp	59,4%	69,1%	71,4%	66,7%
NPV	54,2%	59,2%	38,4%	80%
PPV	74%	79,5%	71,4%	87,5%
Acc	65,9%	75,6%	65,5%	85,7%
F1-score	71,8%	82%	73,7%	90,3%

n: number of exams in the class, Se: sensitivity, Sp: specificity, NPV: Negative Predictive Value, PPV: Positive Predictive Value, Acc: accuracy, Pr: precision, Rec: recall.

### Sleep analysis

A Wilcoxon rank sum test for the difference between median TST of the PSG and of ECG-based detection algorithm was performed as the TST was not distributed normally and gave a statistically significant result with a p-value of.015. ECG-based detection algorithm tended to underestimate TST by 15.6 minutes with limits of agreement from -4.2 to 3.7h. Results are presented in Table 7.

**Table 7:** Bias value of TST between polysomnography and the ECG-based detection algorithm for different classes of: (i) severity of AHI, (ii) reliability index.

		Total dataset			Good reliability (green)			Moderate reliability (orange)		
	Total dataset	0-15/h	15-30/h	≥30/h	0-15/h	15-30/h	≥30/h	0-15/h	15-30/h	≥30/h
n	85	32	26	27	21	15	18	11	11	9
Bias (h)	-0.26	0.51	-0.87	-0.58	0.39	-0.46	-1.13	-0.58	-0.72	-1.27
Limits of agreement (h)	[-4.2 ; 3.7]	[-3.9 ; 4.9]	[-4.8 ; 3.1]	[-3 ; 1.8]	[-2.9 ; 3.7]	[-4.5 ; 3.5]	[-3.4 ; 1.1]	[-2.9 ; 1.8]	[-4.4 ; 3]	[-3.2 ; 0.7]
Standard deviation	2.0	2.3	2.0	1.2	1.71	2.05	1.17	1.2	1.9	1.0

n: number of exams in the class.

For exams with good reliability index, bias is of 23.4 minutes for no or mild SAS, -27.6 minutes for moderate SAS and -67.8 minutes for severe SAS. For exams with moderate reliability index, bias is of -34.8 minutes for no or mild SAS, -43.2 minutes for moderate SAS and of -76.2 minutes for severe SAS. Bias is also computed respectively according to the mechanism of the respiratory events (obstructive or central) and predominance of the type of events (hypopneas if hypopneas>apneas, else apneas). Results are presented in Table 8.

**Table 8:** Bias value of TST between polysomnography and the ECG-based detection algorithm for different classes of: (i) predominance of the type of events (hypopneas or apneas), (ii) main mechanism involved in the majority of apneas genesis (obstructive or central).

	Predominance		Mechanism	
	Hypopnea (HI>AI)	Apnea (AI≥HI)	Obstructive	Central
n	38	15	47	6
Bias (h)	-0.79	-0.56	-0.73	-0.65
Limits of agreement (h)	[-4.41; 2.8]	[-2.7; 1.6]	[-4.1; 2.6]	[-3.4; 2.1]
Standard deviation	1.8	1.1	1.7	1.4

n: number of exams in the class.

## Discussion

This study demonstrated that the proposed method dedicated to the evaluation of obstructive sleep apnea syndrome using nocturnal long-term electrocardiogram signals provided a good ability to detect sleep-related respiratory events. On the complete dataset, the sensitivity was 69.8% and the specificity 59.4% at an AHI threshold of 15 events/hour. Focusing on exams with good reliability and at the same AHI cut-off, the performances improved, giving increased sensibility and specificity at 78.5% and 69.1%, respectively.

Morphology of QRS complex might have an influence on results. As retrospective data was used, there was no homogenization regarding the recorded lead and placement of electrodes, generating monophasic or biphasic QRS complex on DII. Considering good reliability signals, recordings with monophasic QRS complex demonstrated higher sensibility and accuracy and similar specificity compared to recording with biphasic QRS complex, respectively 93.3% vs 63.6%, 85.7% vs 65.5% and 66.7% vs 71.4%. The differences could be explained by the dataset used for the ECG-based detection algorithm training set. Avoiding misplacement and inversion of electrodes seems to be essential to improve the evaluation of obstructive sleep apnea using this method.

Since only one bipolar channel (DII ECG) was recorded, this method falls into the category of type IV sleep devices. Our results showed consistent values with the performance of type IV Home Sleep Apnea Tests (one channel-based sleep recording) respectively varying from 43 to 100% and from 42 to 100% in the literature. To note, type III Home Sleep Apnea Tests used to exhibit performance values varying from 64 to 100% and from 41 to 100% to the studied method. Thus, the proposed method showed an equivalent ability to detect sleep-related breathing respiratory events as type III tests, but without oro-nasal airflow sensor coupled with oximetry. This would be interesting for healthcare professionals to have such a device available, combining type IV-related ergonomics and type III-related performances.

Several clinical studies have already shown that the results of type IV studies were comparable to those of type III studies [23–26]. Other studies have also shown that simplified diagnostic strategies and other models of outpatient care involving trained nurses and/or primary care physicians can produce non-inferior results compared to laboratory tests led by specialists [26–28]. In [29], results of the clinical study confirmed the non-inferiority of the manually scored type III tests but type IV tests provided poorer outcomes. A report from the Agency for Healthcare Research and Quality indicates that, for the diagnosis of OSAHS using type III or IV devices, the higher the threshold for AHI (moderate to severe OSAHS), the better the specificity (with less variability) with type I as reference [30]. More recently, in 2018, a systematic review screened 6,054 abstracts, 117 full-text articles and 10 portable monitors dedicated to home sleep apnea testing and showed that the bias between PSG-measured and estimated AHI ranged from -14.8 to 10.6 events/h. When AHI ≥  5 events/h, the sensitivity of type IV portables monitors ranged from 67.5–100% and specificity ranged from 25 to 100%. In [8], results of 25 papers and 30 commercial devices were reviewed. Among them, no commercial device and only two studies [15,16] tested type IV devices with only one electrocardiogram-type heart sensor to diagnose OSAHS. Data used in those two studies came from the same origin: Physionet Apnea-ECG Database [31] composed of 32 subjects. The quality rating was low since at least three indicators among the ten proposed by Flemons *et al.* [9] did not meet the expected criteria, such as blinded comparisons. Before assessing the performance of the proposed fully automated method on retrospective clinical data, we first evaluated it using the Physionet Apnea-ECG Database. At an AHI threshold of 15 events per hour, the method achieved a sensitivity of 84.21% and a specificity of 85.71%. The performance was improved with this commonly-used database. This difference may be attributed to the higher quality of signals in the Physionet Apnea-ECG Database compared to real-life data used in this study.

The proposed method also discriminated results using a reliability index. This index has been considering as a crucial factor in machine learning and deep learning fields to determine the resemblance with what the algorithm has learned. As mentioned, in this study, signals with a good reliability showed increasing performance values compared to the complete dataset. This parameter appeared as a relevant indicator to evaluate the quality and the reliability of the results.

In the frame of adults with comorbid conditions such as resistant high blood pressure, history of stroke, chronic heart or renal failure, mood disorders and atrial fibrillation, an AHI threshold of 15 pathological respiratory events per hour remains the most relevant cut-off. For these patients, even a moderate obstructive sleep apnea is considered as a pathologic condition and may lead to a therapy. As this method is an aid to the evaluation of sleep apnea syndrome, high-risked population must be properly detected. The ability of detecting sleep apnea on these populations with type III device using among other electrocardiogram has been previously demonstrated [32,33]. Further studies will be required to assess the interest of the proposed method and validate its performances on specific subpopulations.

The diagnosis of sleep apnea syndrome is challenging because of the variety of symptoms and respiratory events. The proposed method succeeded to discriminate obstructive from central apneas, and hypopnea from apnea. The bias value for AHI was higher for central apneas compared to obstructive apneas (12.2 + /- 26.4/h vs 11.3 + /- 21.5/h), demonstrating an increased ability to detect obstructive than central apneas. As mentioned previously, sleep-related breathing disorders induce CVHR but with more pronounced heart rate variations observed during obstructive compared to central apneas. Indeed, obstructive apneas induce repetitive major intrathoracic negative pressures leading to marked variations of stroke volume across respiratory cycles and consequently obvious variations of the amplitude of HR. On the contrary, central-related respiratory events are linked to chronic and constant decreased cardiac output (or rarely linked to altered central chemosensitivity). Periodic respiratory arrests are even more the consequence of chronic cardiac failure than the cause, the first impacting sparsely the latter, with a lesser effect on CVHR. Similar limits explain a higher bias for AHI for hypopneas compared to apneas (14.9 + /-26.4/h vs 10.9 + /-25.5/h). In the case of obstructive-related hypopneas, the inspiratory efforts are reduced compared to obstructive apneas, so is the stroke volume variations across respiratory cycles and consequently the amplitude of HR variations. A lesser variation of amplitude of HR makes the detection of respiratory events more difficult, increasing the bias if hypopneas represent the majority of respiratory events. In the case of central-related hypopneas, the ability of the algorithm to detect the respiratory event remains uncertain, because of a pattern of the event very close to physiologic CVHR. All-in-one, CVHR is even more impacted in case obstructive events, especially obstructive apneas. In the same way, it has been demonstrated that the presence of such CVHR peaks 30% of the time has been shown to be associated with a positive predictive value of > 90% in identifying severe OSAHS, (AHI ≥ 30/h) [34]. Since the proposed ECG-based detection algorithm is partly based on HR, obstructive sleep apnea seems to be the best detectable respiratory event. This profile of performance for detecting obstructive apnea highlighted two points: (1) in case of a majority of hypopnea, or majority of central apnea, bias might be higher than expected, and (2) the use of this device should focus on patients referred for OSAHS and not for Central Sleep Apnea-Hypopnea Syndrome (CSAHS).

Type IV devices have been developed for home recordings and are expected to be user-friendly both for patients and clinicians, with generally lower costs, thus easily implementable in a wide care population. Compared to conventional type III devices, the evaluation of sleep apnea from nocturnal long-term electrocardiogram signals avoids the application of a nasal canula and pulse oximetry. This is a major concern because these sensors can be source of data lost and patient discomfort. The peribuccal region and the extremities of fingers, where nasal canula and pulse oximetry are respectively placed, are very nerve sensitive areas. Any stimulus may become rapidly discomfortable and awakening. Using only nocturnal long-term electrocardiogram signals may increase the patient comfort while allowing prolonged monitoring as continuous nighttime and daytime recording. Considering this, the proposed method offers a simplicity of use for clinician and a well-expected adhesion by the patient inherent to type IV devices, but with the performances of type III, making it highly competitive. It would be a very useful tool for healthcare professionals to evaluate sleep in patients at risk of moderate or severe sleep apnea at home. In the protocol of this study, the algorithms’ results were blindly compared to the gold standard, polysomnography reviewed and scored by a trained physician, without any reviewing by a healthcare professional for the algorithms. As those are part of a cloud-based medical device, the performance of the entire device would have to be evaluated in real conditions in the future. This would aim to assess the evaluation of sleep-related breathing disorders after proofreading of the results by a physician.

In conclusion, the proposed method for evaluation of obstructive sleep apnea using nocturnal long-term electrocardiogram signals showed high performances, close to type III devices for preliminary evaluation of patients suspected or at-risk of OSAHS. We identified two key points that could improve performances of the algorithm: (1) a preliminary identification of the cardiac electrical axis related to the patient, to adapt the placement of ECG electrodes and optimize the monophasic aspect of QRS, and (2) the use of the reliability index selecting high quality ECG signal before processing apnea detection. Now, further studies are needed to measure the performance of this method for evaluating OSAHS with patients in real home conditions and with proofreading of the results by a healthcare professional.

## Abbreviations

AASM: American Academy of Sleep Medicine
AF: Atrial fibrillation
AHI: Apnea-Hypopnea Index
AI: Apnea Index
BMI: Body Mass Index
CPAP: Continuous Positive Airway Pressure
CSAHS: Central Sleep Apnea-Hypopnea Syndrome
CVHR: Cyclic Variation of Heart Rate
ECG: Electrocardiogram
EDR: ECG-Derived Respiration
EEG: Electroencephalogram
EMG: Electromyogram
EOG: Electrooculogram
HI: Hypopnea Index
HR: Heart Rate
HRV: Heart-Rate Variability
OMP: Orthogonal Matching Pursuit
OSAHS: Obstructive Sleep Apnea-Hypopnea Syndrome
PSG: Polysomnography
RWA: R-Wave Amplitude
SAS: Sleep Apnea Syndrome
TST: Total Sleep Time
