# Correction: Robust performances of a nocturnal long-term ECG algorithm for the evaluation of sleep apnea syndrome: A pilot study

**DOI:** 10.1371/journal.pone.0332320

**Published:** 2025-09-11

**Authors:** Pauline Guyot, Morgane Eveilleau, Thierry Bastogne, Carole Ayav, Nicolas Carpentier, Bruno Chenuel

The images for Figs 1 to 3 and 5 to 7 are incorrectly switched. The image that appears as Fig 1 should be Fig 7, the image that appears as Fig 2 should be Fig 5, the image that appears as Fig 3 should be Fig 6, the image that appears as Fig 5 should be Fig 3 and the image that appears as Fig 6 should be Fig 2. The figure captions appear in the correct order. The authors have provided a corrected version of figures here.

**Fig 1:**
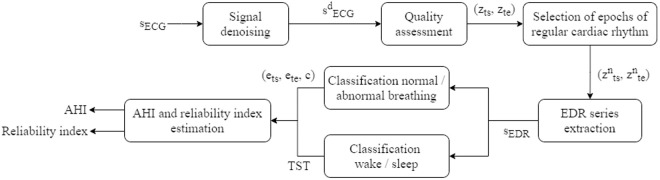
ECG lead-based algorithm diagram. sECG: raw ECG signal, s^d^ECG: denoised ECG signal, (z_ts_, z_te_): start and end of good quality signal zones, (z^n^_ts_, z^n^_te_): start and end of good quality signal combined with normal cardiac rhythm zones, ^s^EDR: EDR series, (e_ts_, e_te_, c): start, end and class of abnormal events (apnea or hypopnea), TST: total sleep time, AHI: apnea-hypopnea index.

**Fig 2:**
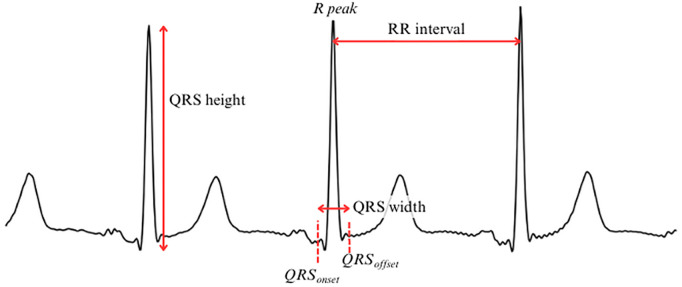
Features of interest detected or computed on an ECG signal. R peak, QRS_onset_ and QRS_offset_ are key points in an ECG beat, whereas QRS height, QRS width and RR interval are computed using the previous key points.

**Fig 3:**
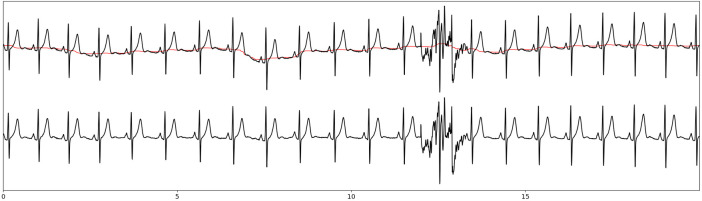
Signal denoising (baseline correction and powerline noise removal) example. Top: raw ECG signal in black with baseline wander in red; down: denoised ECG signal. X-axis is the time scale in seconds.

**Fig 5:**
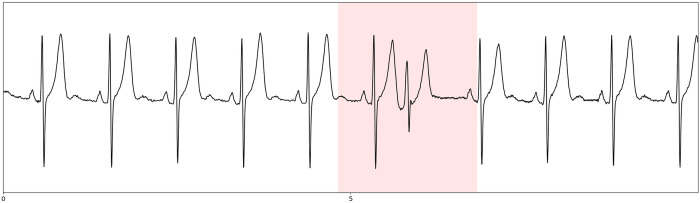
Detection of an abnormal beat example. In red, an abnormal beat zone to be suppressed for the rest of the analysis. X-axis is the time scale in seconds.

**Fig 6:**
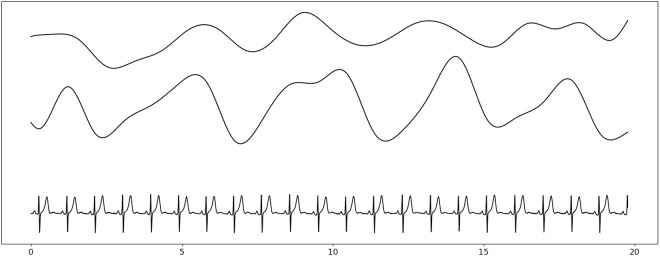
Example of EDR series extracted from ECG signal. From top to bottom: HRV series, RWA series and denoised ECG signal. X-axis is the time scale in seconds.

**Fig 7:**
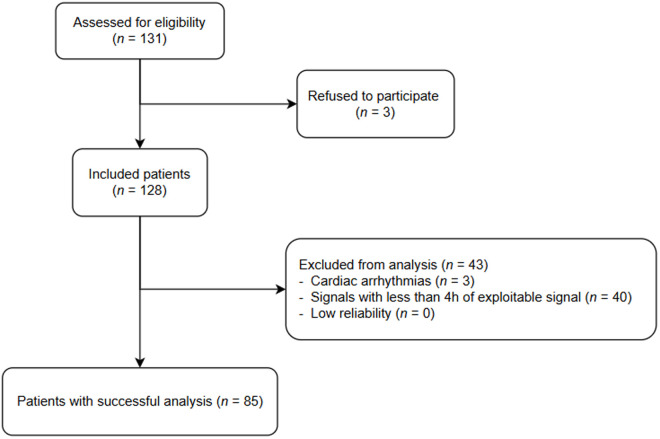
Flow chart of study. n: number of patients.
